# Alpha-SGANet: A multi-attention-scale feature pyramid network combined with lightweight network based on Alpha-IoU loss

**DOI:** 10.1371/journal.pone.0276581

**Published:** 2022-10-27

**Authors:** Hong Li, Qian Zhou, Yao Mao, Bing Zhang, Chao Liu

**Affiliations:** 1 Institute of Optics and Electronics, Chinese Academy of Science, Chengdu, China; 2 Key Laboratory of Optical Engineering, Chinese Academy of Science, Chengdu, China; 3 University of Chinese Academy of Science, Beijing, China; Hanyang University, REPUBLIC OF KOREA

## Abstract

The design of deep convolutional neural networks has resulted in significant advances and successes in the field of object detection. However, despite these achievements, the high computational and memory costs of such object detection networks on the edge or in mobile scenarios are one of the most significant barriers to their broad adoption. To solve this problem, this paper introduces an improved lightweight real-time convolutional neural network based on YOLOv5, called Alpha-SGANet: A multi-attention-scale feature pyramid network combined with a lightweight network based on Alpha-IoU loss. Firstly, we add one more prediction head to detect different-scale objects, design a lightweight and efficient feature extraction network using ShuffleNetV2 in the backbone, and reduce information loss using the SPP module with a smaller convolutional nucleus. Then, cleverly, employ GAFPN to improve feature transition processing in the neck region, including the usage of the Ghost module to construct efficient feature maps to help prediction. The CBAM module was further integrated to find areas of interest in the scene; finally, combined with Alpha-IOU loss for model supervision training, the biggest performance improvement was achieved. The experiment results show that, compared with YOLOv5s, our proposed method can achieve higher accuracy with fewer parameters and has real-time speed through verification on the PASCAL VOC dataset and MS COCO dataset.

## Introduction

One of the most critical problems in computer vision is object detection, which involves the localization and classification of multiple objects in an image or video. Object detection has a wide range of applications, including human posture detection [1], video surveillance [2], unmanned autonomous vehicles [3], face detection [4], and so on. The rapid development of deep learning has spawned new research in the field of object detection, and various related object detection frameworks have emerged also and become mainstream. Due to its complicated structure and a significant amount of processing, the object identification approach based on deep learning offers good performance, but it is difficult to run and deploy in real time on platforms such as embedded and mobile terminals. Deep learning’s performance in practical applications has become increasingly important to researchers.

In the past few years, the two-stage target detection algorithm was developed to increase target identification accuracy by first producing a large number of candidate target frames and then carefully screening them. However, this method was extremely slow due to the fixed detection mode’s two-step division, such as RCNN [5], Fast RCNN [6], and Faster RCNN [7]. One-stage object detection methods predict object boxes by direct regression, this is a simplified method from the overall steps of detection, such as YOLO [8] and SSD [9]. This approach can greatly speed up inference and is more suited to object detection in real-time applications, but it still has severe drawbacks. It primarily focuses on the detection process’s simplification and ignores the network structure’s complexity, resulting in an increase in network parameters and massively increased computational needs. This makes deploying cutting-edge CNN models on resource-constrained platforms like embedded and mobile devices problematic.

People have proposed a series of approaches to explore compact neural networks, such as network pruning [10], low-bit quantization [11], knowledge distillation [12], and other methods of model compression, to tackle the challenges and adapt to the real-time scenario requirement. Besides, designing deep neural network architecture for the optimal trade-off between accuracy and efficiency has been an active research area in recent years. Both novel handcrafted structures and algorithmic neural architecture search have played important roles in advancing this field.

The problem of developing efficient CNN architectures for offering high-quality services on edge devices has gotten a lot of attention recently. This technique has had a lot of success and has presented a lot of new architectures. e.g., MobileNet [13–15], ShuffleNet [16, 17], GhostNet [18], etc. The object detection method combined with a lightweight network is more suited to edge platforms, as it may successfully reduce model parameters while maintaining network accuracy. To achieve real-time object detection on mobile platforms with limited computing resources. In this paper, an improved lightweight real-time object detection method Alpha-SGANet is proposed by combining YOLOv5s with a lightweight network. Firstly, considering the cost-effectiveness, we add one more prediction head to detect different-scale objects, that is, increase the P6 downsampling layer to increase the receptive field of the network, and build a lightweight and efficient feature extraction network using ShuffleNetV2 in the backbone part, and also use SPP module with a smaller convolutional nucleus to suppress information loss; Then to further increase the detection performance of the network, we make improvements to the neck part, proposing a variant of PAFPN [19] named GAFPN to help feature transition processing, cleverly uses the Ghost module to generate efficient feature maps to help predict, and further integrates the CBAM [20] module to find areas of interest in the scene; Finally, combined with Alpha-IoU loss [21], the model supervised training obtained the largest performance improvement. Experimental results demonstrate that our proposed real-time object detection method Alpha-SGANet achieves the best accuracy performance on the PASCAL VOC dataset and MS COCO dataset, and its speed is real-time. To better balance the speed and accuracy, the proposed small model version Alpha-SGAsNet has greatly improved the accuracy compared with the original YOLOv5s in the case of a slight decrease in speed, and it realizes the lightweight of the model. It can be easily deployed on embedded platforms and edge mobile devices, and it has better real-time performance after the embedded platform is verified.

**Fig 1:**
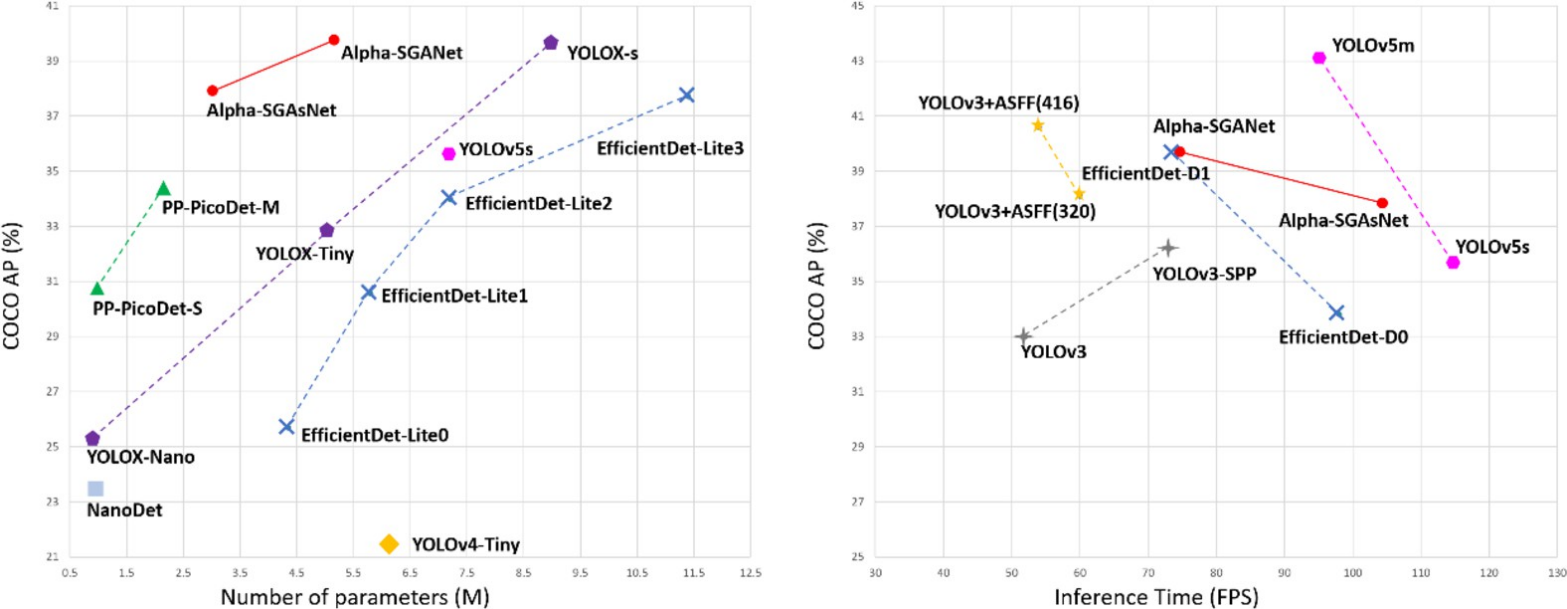
Speed-accuracy trade-off of accurate models (top) and Size-accuracy curve of models for Alpha-SGANet and other state-of-the-art object detectors.

With the practical updates of the aforementioned strategies, as represented in Fig 1, we boost the YOLOv5s to 139.73% mAP on COCO with 640×640 the resolution, Compared with the current optimal network models including PP-PicoDet [22] and YOLOX [23], Alpha-SGANet has certain competitiveness in terms of model parameters and inference speed and has good performance. The proposed small model version Alpha-SGAsNet has the best performance in the trade-off between speed and accuracy. Compared with the original YOLOv5s, the accuracy is greatly improved with only a slight decrease in speed.

To summarize, our main contributions are as follows:

(1) For embedded platforms and mobile devices, a lightweight real-time target detection network Alpha-SGANet is proposed, which has significantly improved detection accuracy.
(2) The proposed small model version Alpha-SGAsNet can better balance the speed and accuracy issues, realize the lightweight of the model, and verify that it has better accuracy and speed performance on the embedded platform.

The remainder of this work is arranged in the following manner. Section 1 introduces the object detection and challenges and some methods of real-time object detection in embedded platforms or mobile devices. Some methods of object detection and model lightweight, and some FPN-related work are introduced in detail in Section 2. We elaborate on the details of our Alpha-SGANet in Section 3. Experimental results, analyses, and comparisons are given in Section 4, and Section 5 provides concluding remarks and future work.

## Related work

Most of the traditional object detection methods are based on manual feature design methods, such as HOG (Histogram of oriented gradient) [24] and Scale-invariant feature transform (SIFT) [25], etc. compared to traditional methods, The method is based on a neural network has a more essential characterization of image features and has a strong expressive ability. The rapid development of deep learning has also promoted the development of object detection. From the classic DPM algorithm [26] to the RCNN [5], the pioneer of deep learning target detection, to the Faster-RCNN [7], and on to the YOLO series, culminating in the YOLOX [23] in recent years, and so on, Researchers are constantly pursuing the best feasible improvement speed while ensuring accuracy, from advanced optimization of the two-stage algorithm to single-stage algorithm, from Anchor-based approach to Anchor-Free method.

### Real-time object detection

The object detection problem is treated as a regression task by the one-stage detector, which eliminates the requirement for a complicated pipeline. This is also why the one-stage target detection method outperforms the two-stage target detection method in terms of speed. The audience is also aware of the real-time consequences of the YOLO series and SSD series methods on GPU. The most representative one-stage technique from input to output end-to-end is proposed by YOLOv1 [8]. The basic concept is to divide the image into *s*×*s* grids, each of which predicts a different target. SSD [9] predicts with multi-scale features and performs better than YOLOv1 in terms of speed and accuracy. RetinaNet [27] proposes a better version of SSD to overcome the problem of unbalanced positive and negative instances, based on the concept of Focal Loss. Faced with the issue of unbalanced positive and negative samples, ATSS [28] developed the definition of positive and negative samples as well as the ultimate effect of selection on the model, allowing RetinaNet to be enhanced further by adaptively picking positive samples. Multi-scale training, adaptive previous frame, and other YOLOv1-based techniques are used to improve YOLOv2 [29]. For the first time, YOLOv3 [30] introduced the revolutionary DrakNet53 as the backbone network for feature extraction and attempted the idea of FPN to detect on three feature sizes for the first time. It provides the best performance in terms of speed and accuracy because to its unique network structure design. YOLOv4 [31], YOLOv5, and other works based on YOLOv3 improve the trade-off between speed and accuracy by combining the advantages of numerous algorithms and some improvement talents. With detection head decoupling and the SimOTA label assignment technique, YOLOX [23] produces state-of-the-art outcomes in a large variety of models. FCOS [32] proposes a one-stage full convolution object detector based on pixel-level prediction. Through model scaling and neural architecture search, EfficientNet [33] discovers the optimal combination of network depth, network width, and input image resolution. Scaled-YOLOv4 [34] recommends that in practical engineering deployment, multiple models be designed using model scaling for different platforms and then compared. Some keypoint-based techniques, such as CornerNet [35], CenterNet [36], and CentripetalNet [37], have also piqued the interest of academics.

### Lightweight network

Some scholars have offered model compression approaches as a solution to this challenge, such as pruning, quantization, knowledge distillation, and low-rank decomposition. Another group proposed to explore the network structure deeply, such as EfficientNet [33] to find a suitable relationship between input resolution and network width/depth. Besides them, efficient neural architecture design has a very high potential for establishing highly efficient deep networks with fewer parameters and calculations and recently has achieved considerable success. SqueezeNet [38] achieves model compression by stacking fire modules including squeeze and expand. MobileNet [13–15] utilized the depthwise and pointwise convolutions to construct a unit for approximating the original convolutional layer with larger filters and achieve comparable performance. ShuffleNet [16, 17] further explored a channel shuffle operation to enhance the performance of lightweight models. GhostNet [18] introduces a novel Ghost module to generate more features by using fewer parameters. Sandglass [39] propose to file the structure and present a novel bottleneck design, called the sandglass block, that performs identity mapping and spatial transformation at higher dimensions and thus alleviates information loss and gradient confusion effectively. Combining the lightweight network as the backbone network for feature extraction can effectively ensure accuracy while greatly reducing network parameters. In this paper, ShuffleNetV2 is used as the backbone network to accelerate the network. Besides, to further improve the detection performance of the network, it is also necessary to design a reasonable neck part, that is, to improve the FPN of the neck part.

### Feature pyramid network

High-level feature maps have a broader receptive field, rich in semantic information about objects, whereas low-level feature maps have more detailed information for multi-scale feature maps. To effectively integrate the information of these two parts to achieve more accurate detection, we often build an FPN architecture in the Neck part to fuse information at multiple scales, so that the network takes into account both the semantic information of high-level features and the detailed information of low-level features, helping improve the detection performance of the network. FPN [40] builds a feature pyramid by sequentially combining two adjacent levels of features with top-down pathway and lateral connections. Such connections effectively enhance feature representations and the rich semantics from deep and low-resolution features are shared at all levels. PANet [19] proposes an additional bottom-up pathway based on FPN to increase the low-level information in deep layers. BiFPN [33] simplifies PANet, proposes a new cross-scale connection method, and assigns different scale feature weights for fusion. Libra-RCNN [41] also integrates features at all levels to generate more balanced semantical features. A pyramid enhancement module is proposed in PAGE-Net [42], which effectively helps to improve the performance of the model by stacking attention mechanisms of different scales to obtain features with larger receptive fields. In addition to manually designing the fusion structure, NAS-FPN [43] applies the Neural Architecture Search algorithm to seek a more powerful fusion architecture, delivering the best single-shot detector. Different from the way that NAS-FPN uses the neural network architecture to automatically search, our method is to manually design the FPN architecture, and propose a variant of PAFPN called GAFPN, which mainly uses the Ghost module and the dual-channel attention mechanism CBAM [20]. GAFPN can effectively improve the detection performance of the network with little or no increase in the amount of computation.

## Method description

This section provides a thorough overview of the neural network framework that has been suggested. Our primary objective is to enable real-time target detection for platforms with limited resources, such as embedded and edge mobile devices. How to meet real-time requirements while maintaining accuracy is a speed-accuracy trade-off conundrum. YOLOv5 is one of the most recent object detection algorithms, with the most advanced object detection performance, to get the best benefits of the two. Based on the network topology of YOLOv5s, we propose the Alpha-SGANet object detection technique. This section introduces the overall network design before delving into the various components of our network.

### Overall architecture

**Fig 2:**
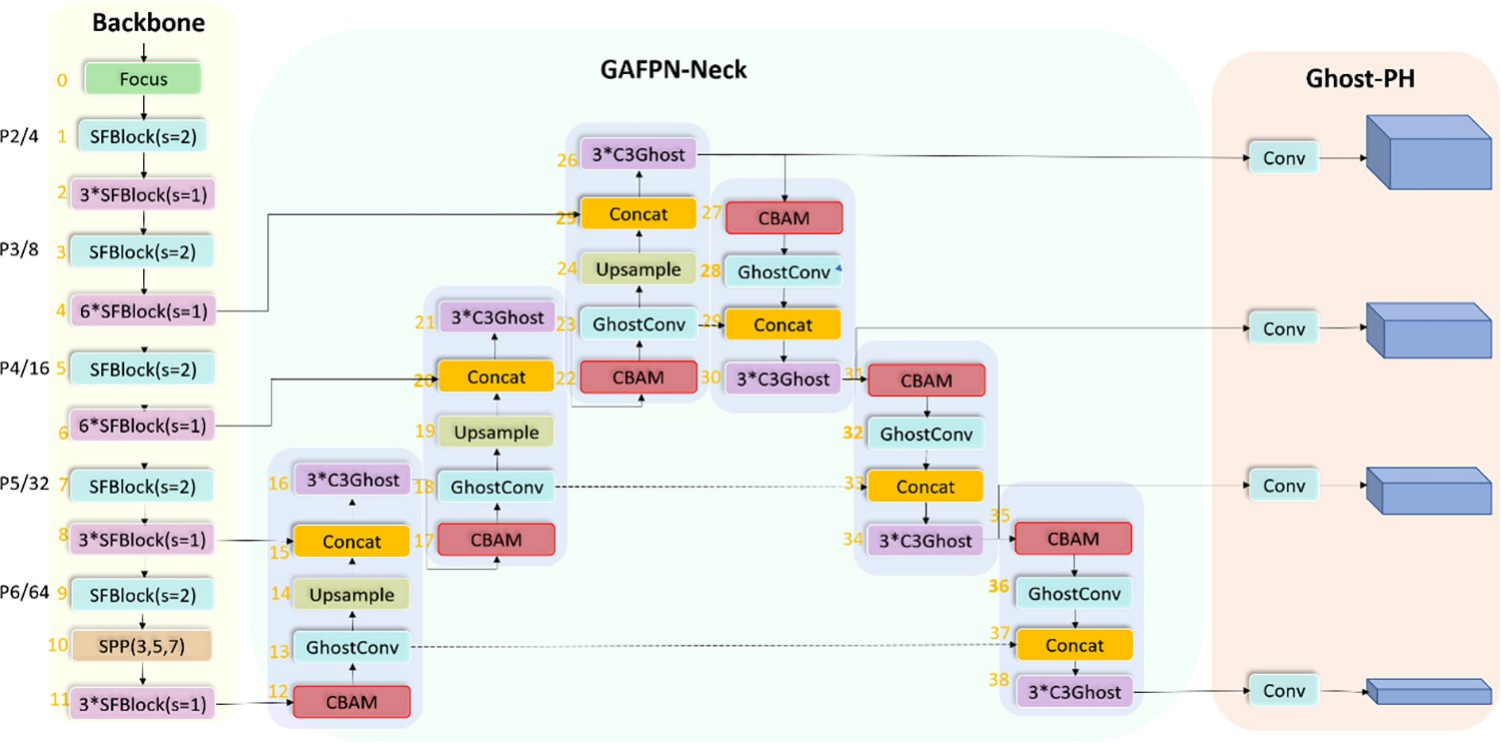
An overview of the Alpha-SGANet framework is demonstrated. The method contains a total of three parts: ShuffleNetV2 Backbone, GAFPN Nect, and Detection Head. The backbone part uses ShuffleNetV2 modules to build a lightweight and efficient feature extraction network; GAFPN Neck helps feature processing transition; finally Detection Head part uses the Ghost module to generate a rich feature map to help guide the prediction of the target frame.

As illustrated in Fig 2, our proposed Alpha-SGANet consists of three sections: ShuffleNetV2 Backbone, GAFPN-Neck, Ghost-PH, As we can see, we have mainly improved the Backbone part and the Neck part. Firstly, to reduce model parameters and achieve lightweight, a lightweight ShuffleNetV2 [17] module is used to build a lightweight and efficient feature extraction network in the backbone network, and we perform one more downsampling to detect at four feature scales, namely the 64-fold downsampling layer of P6 is added to increase the receptive field of the network. Then, to further improve the detection performance of the model, an attention mechanism-guided variant of PAFPN, GAFPN, is proposed to further process the feature map, in which CBAM [20] applies weights from both space and channel to screen out favorable spatial information and channel feature information to help Model learning of features. It can be noted that to meet the requirements of lightweight design and also help feature processing, we also use the C3Ghost basic unit constructed by the lightweight network Ghost bottleneck in the neck part. In the beginning, our starting point was only to reduce the number of parameters of the model and accelerate the acceleration of model inference, so at the beginning, we only tried to combine the lightweight network in the backbone part, but we found that using C3Ghost at the same time in the backbone and neck parts would greatly improve the performance of the model, but the speed drop is also obvious. To improve the speed, I tried to combine the lightest ShuffleNetV2 module in the backbone part, and the speed was improved when the accuracy was slightly reduced. And note that after the second downsampling (P2), we start to repeat the stacking of the ShuffleNetV2 module with a stride of 1. To fully extract the features, we then perform the third (P3) and fourth downsampling (P4) and then repeated stacking 6 times, the purpose of which is to fully extract features and generate more effective feature maps.

After the fifth downsampling (P5) and the sixth downsampling, only 3 repeated stackings are used, which is also to take into account that the number of deeper channels increases, which leads to an increase in the amount of calculation. It will cause the load on the inference calculation process of the model and affect the real-time inference of the model. It should be noted that for the SPP here, we choose to use a smaller receptive field range and choose a maximum pooling convolution kernel such as {3, 5, 7}, which also contributes to the final result.

In addition, we try to replace the original DIoU Loss with the latest work Alpha-IoU Loss [21], so the proposed network is called Alpha-SGANet. The contribution of Alpha-IoU Loss to our final model is huge, the effect of improving the accuracy of the model is obvious, and the improvement effect of Alpha-IoU Loss on the lightweight network is better during the experiment. Finally, our model Alpha-SGANet achieves the best accuracy of 65.14% mAP on the VOC dataset with a parameter size of 4.93 MB, and the real-time speed can reach 68.49 FPS. On the COCO dataset, the accuracy has 39.73% mAP and the real-time speed has 74.6 FPS. To further take into account the best balance between speed and accuracy, we also propose a small model version of the network called Alpha-SGAsNet, that is, there is no P6 layer, only three feature scales are detected, and finally it is on the VOC dataset. The parameter volume of 2.84 MB obtains an accuracy of 62.62% mAP, its real-time speed has 105.3 FPS, and it obtains 37.84% mAP on the MS COCO dataset with a parameter volume of 3.01 MB, and the real-time speed has 104.2 FPS, which is good for embedded platforms and edges The contribution of end-to-end real-time object detection is significant.

### ShuffleNetV2 backbone

**Fig 3:**
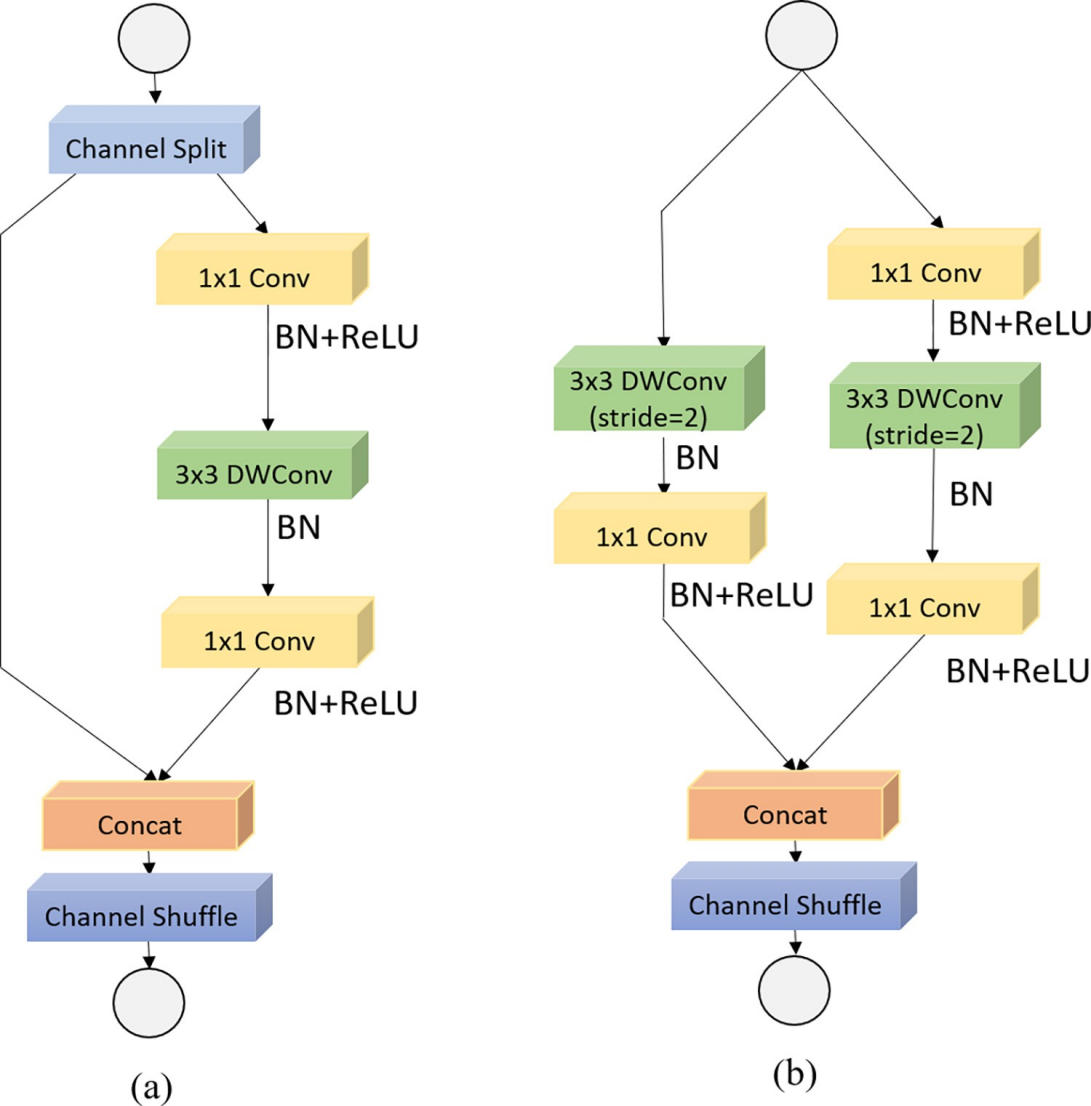
Building blocks of ShuffleNetV2. (a) the basic ShuffleNetV2; (b) the ShuffleNetV2 unit for spatial down sampling(2x). DwConv depthwise convolution.

We concluded that ShuffleNetV2 [17] is the most adaptable and has the greatest performance on mobile devices depending on multiple experiments. The number of float-point operations, or FLOPs, is a commonly used indicator for evaluating computer complexity; the specific representation is the number of multiply-add operations. FLOPs, on the other hand, are an indirect metric. It is a close approximation of the direct measure we care about, such as speed or latency, although it is rarely equal. Because model inference speed is affected by more than simply FLOPs, FLOPs ignore several crucial aspects that have a significant impact on speed. Memory access cost (MAC) is one such component that could be a bottleneck on devices with high processing capability, such as GPUs. The degree of parallelism is another. Under the same FLOPs, a model with a high degree of parallelism could be substantially faster than one with a low degree of parallelism. In 2018, ShuffleNetV2 was proposed as a useful categorization network. Simultaneously, its team blended theory and experiment, resulting in four practical recommendations: (1) The MAC is the smallest and the model speed is the fastest when the number of input and output feature channels of the convolutional layer are equal. (2) The MAC will rise significantly of the convolution group operation, decelerating the model. When there are a lot of groups, it’s best not to employ group convolution. (3) The model speed increases as the number of branches in the model decrease. Some models, like Inception, have a "multi-path" topology, which can easily split the network, restrict model parallelism, and result in slower speeds. (4) Element-wise operations consume substantially more time than the value shown in FLOPs, hence they should be avoided as much as possible. The authors studied the flaws of the ShuffleNetV1 design and refined it based on the four criteria listed above to generate ShuffleNetV2. The module structure of ShuffleNetV2 is shown in Fig 3. There are two types according to the step length: Fig 3(A) the basic ShuffleNetV2, and *s* = 2. sin Fig 3(B) the ShuffleNetV2 unit for spatial downsampling(2x). In this paper, we use the ShuffleNetV2 module to build a lightweight and efficient feature extraction network with stacking times of {3, 6, 6, 3, 3}, respectively, and use the ShuffleNetV2 module in the middle. Downsampling is performed, hence the name ShuffleNetV2 Backbone.

### GAFPN

After trying to combine the lightweight network, the size of the model and the number of parameters have been greatly reduced, and the model inference speed has also been improved, but faced with the problem of decreased detection accuracy. At this time, it is necessary to improve and optimize the feature fusion processing part of the neck in combination with the structural features of the model, that is, the optimization of FPN. In this paper, a PAFPN variant named GAFPN is proposed to help the model performance improve. GAFPN can effectively help the model to improve the detection accuracy with a small number of parameters or almost no increase in the number of parameters. As can be seen from Fig 2, GAFPN also follows the design concept of PAFPN, including two top-down and bottom-up feature fusions, and chooses to detect on four feature scales to fully integrate multi-scale feature information. The C3Ghost module generated by the GhostNet module is used to generate more informative feature maps to guide the prediction of the target box, and the CBAM module is further integrated to find the region of interest in the scene. In addition, it should be noted that the GhostConv module is also included in GAFPN. We use GhostConv to change the number of feature channels and perform downsampling operations.

#### Ghost block

**Fig 4:**
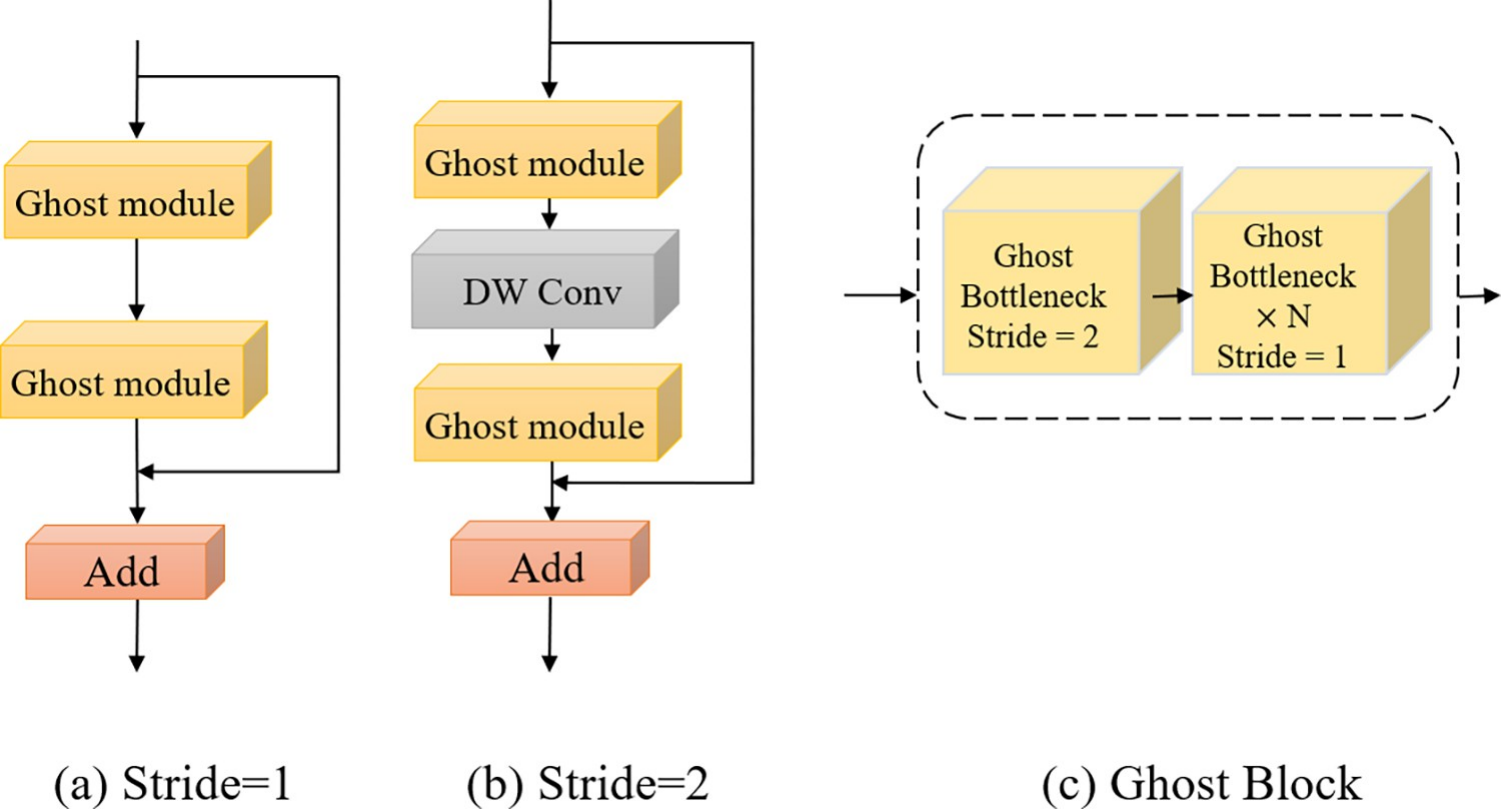
(a) The structure of GhostBottleneck when stride = 1. (b) The structure of GhostBottleneck when stride = 2. (c) The structure of Ghost Block.

It is required to first understand the Ghost Block unit in order to use the C3Ghost module in the GAFPN-Neck section, as shown in Fig 4(C). The Ghost Block is primarily made up of Ghost Bottlenecks, which are classified into two varieties based on their step size. As seen in Fig 4, the Ghost Block module is made up of a Ghost Bottleneck and numerous Ghost Bottleneck connections (c). In this paper, the Ghost Block is simply a stack of Ghost Bottlenecks with a stride of 1. Furthermore, C3Ghost allows numerous Ghost Blocks to be reused, although the number of intermediate channels is governed by the number of output channels.

**Fig 5:**
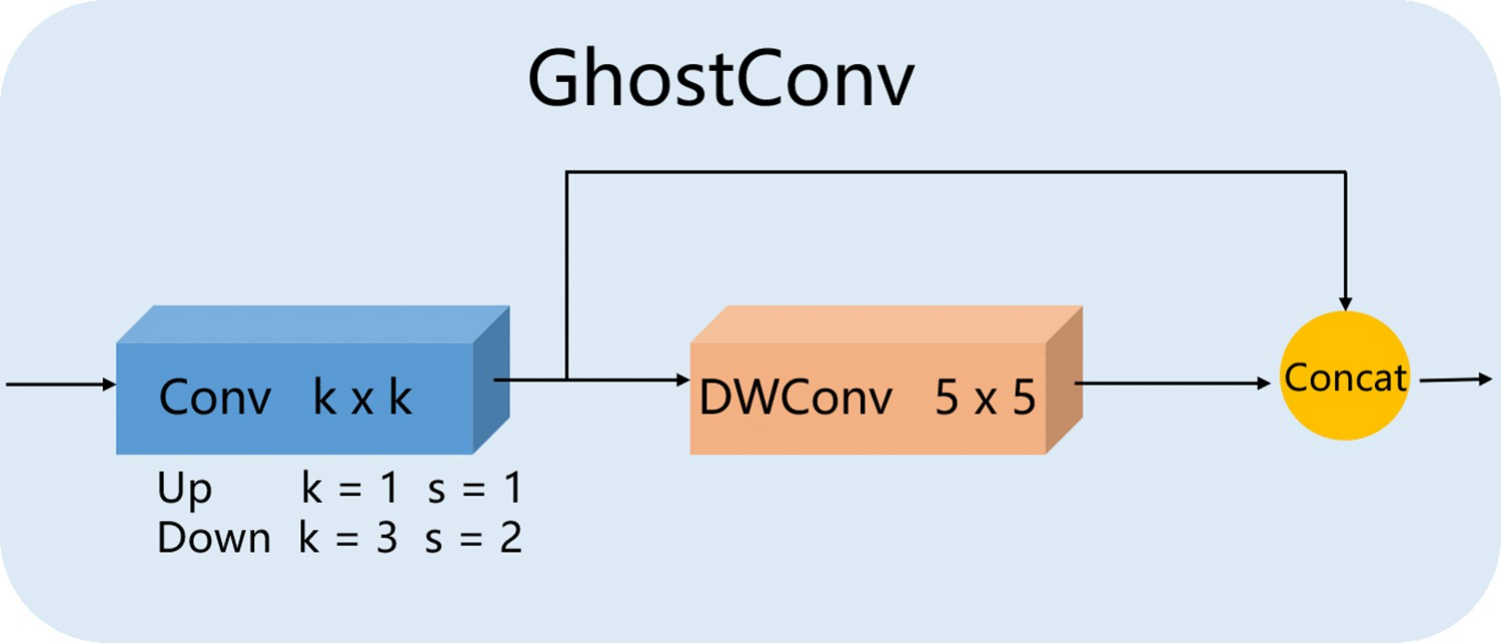
The structure of GhostConv module. Upsampling: *k* = 1, *s* = 1, downsampling: *k* = 3, *s* = 2.

We also built the GhostConv module, which supports feature processing during the upsampling and downsampling operations to create rich feature maps, using the GhostNet concept. Fig 5 depicts the major structure. During the up-sampling process, set the convolution kernel size *k* = 1 and step *s*= 1, During downsampling, set the convolution kernel size *k* = 3 and step *s* = 2 perform downsampling. It’s worth noting that we utilize a convolution kernel of size 5×5 when doing depthwise convolution here, which is also to expand the local range and capture more receptive field information.

#### Ghost module

**Fig 6:**
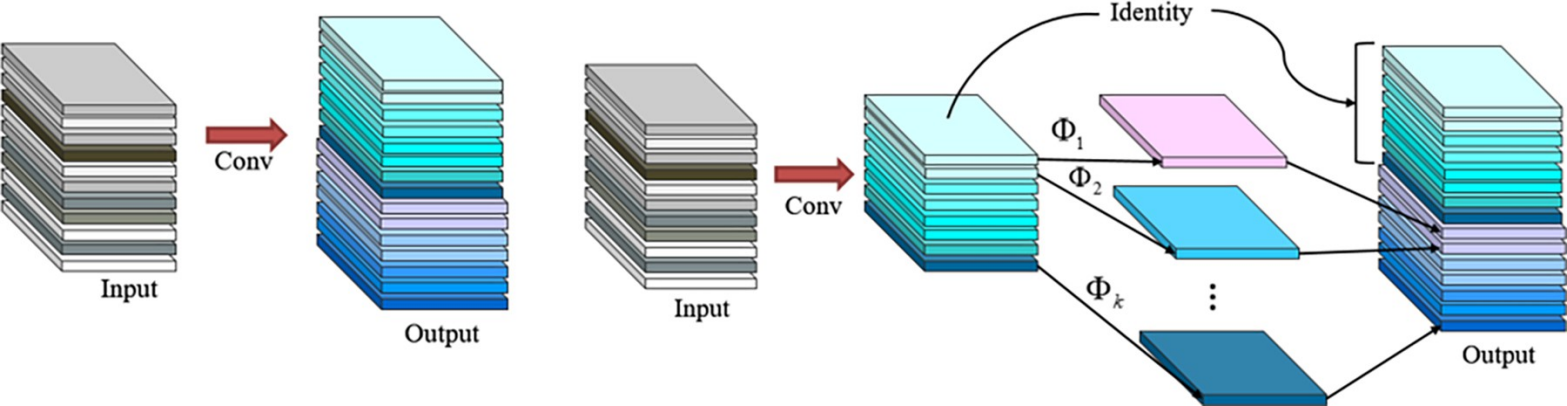
Comparison of Ghost convolution and traditional convolution.

Unlike ordinary convolution, the Ghost module [18] uses a sequence of low-cost linear transformations (Φ_*i*_,*i* = 1,2,…,*k*) to build additional feature maps from low-cost operations. The standard convolution method’s problem of feature redundancy is eliminated, and the quantity of work is considerably decreased. Fig 6 shows its schematic representation in comparison to standard convolution.

Fig 6 shows how the Ghost module generates more features with fewer parameters. A traditional convolutional layer will be split into two sections in deep neural networks. In the first section, ordinary convolutions will be employed, but the total number of them will be rigorously limited. To generate more feature maps, a series of simple linear processes are applied to the intrinsic feature maps from the first section. The Ghost module’s overall parameter count and computational difficulty have been lowered without increasing the size of the output feature map. By simply replacing the convolutional layer with the Ghost module as the convolutional operation in the present network topology, the same feature map may be created at a lower computational cost.

#### CBAM attention module

**Fig 7:**
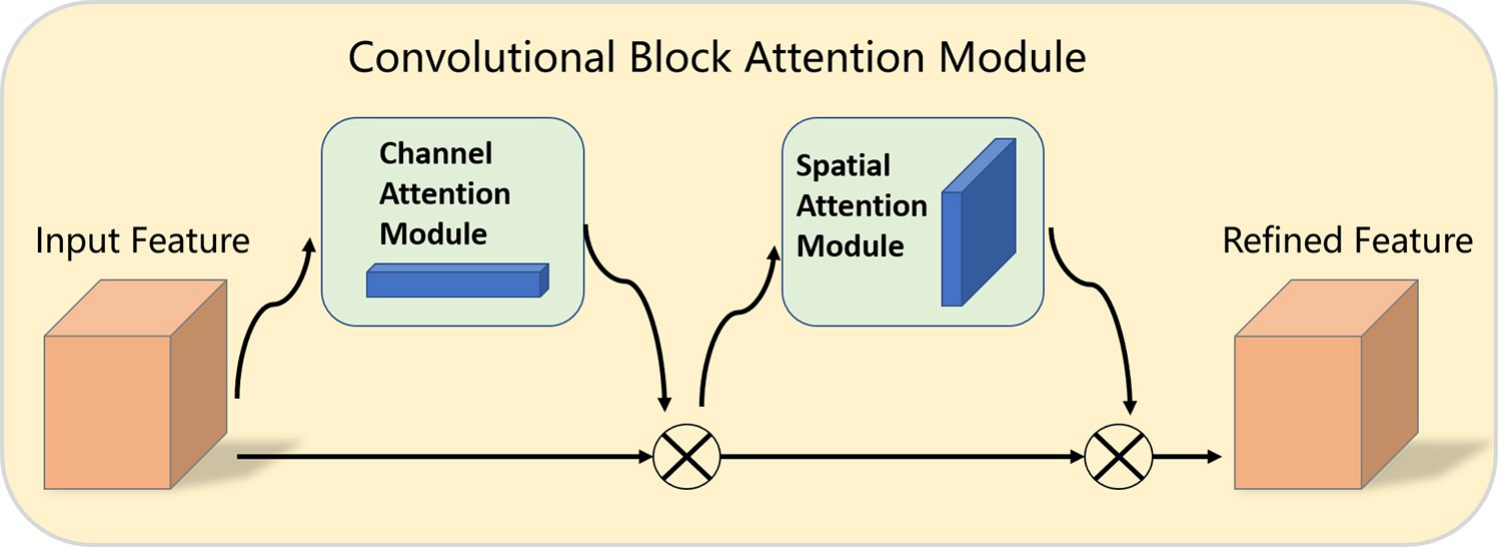
The overview of the CBAM module. Two sequential submodules are used to refine feature maps that go through CBAM, residual paths are also used.

CBAM [20] is a straightforward but powerful attention module. It’s a little module that can be plugged into the most popular CNN architectures and trained in tandem with the basic network. Given a feature map, CBAM infers the attention map in two dimensions: channel and spatial, then multiplies the attention map with the input feature map to accomplish adaptive feature refinement. The CBAM module structure is shown in Fig 7. Experiments show that including CBAM into a variety of models with various classification and detection datasets significantly enhances the model’s performance, demonstrating the module’s utility.

We can simply understand that by using the “sigmoid” method, the original feature map is used to generate the weights of the corresponding dimensions in the channel and space, respectively. These values are a value between 0 and 1, and then the original feature maps of Multiplying, so that the network can pay more attention to more important feature information.

#### Alpha-IoU loss

Early works employ bbox regression losses *L*_*n*_−*norm*, that have already been proved to be sensitive to different bbox scales. Because IoU is the measure for localization and is scale-invariant, recent work has replaced them with the IoU loss and its variations such as GIoU [44], DIoU [45]and CIoU [46] for box regression. The IoU loss is invariant to box scales, unlike *L*_*n*_−*norm* losses, which makes it easier to train better detectors. When the predicted boxes do not match with the ground truth, the IoU loss suffers from the gradient vanishing problem, which slows convergence and leads to incorrect detectors. Convergence is delayed, and detectors are imprecise as a result. This has led to the development of new IoU-based losses such as Generalized IoU (GIoU) [44], Distance-IoU (DIoU) [45], and Complete IoU. (CIoU) [46]. To avoid the gradient vanishing problem, GIoU adds a penalty word to the IoU loss, whereas DIoU and CIoU use penalty terms to account for the central point distance and aspect ratio between predicted boxes and their ground truth. The author introduces the power transformation into the existing IoU Loss, that is, applies the Box-Cox transformation to the IoU loss, generalizes it to a more general form, and adds power regularisation term, called Loss, which has the advantages of existing IoU-based loss functions to obtain more accurate bounding box regression and object detection accuracy. The vanilla IoU loss is defined as *L*_*IOU*_ = 1−*IoU* the author first applying the Box-Cox transformation and generalizing the IoU loss to a loss:

$$L_{\alpha -IoU}=\frac{1-IoU^{\alpha }}{\alpha },\alpha >0$$ (1)

Most of the IoU terms in existing losses, such as *log*(*IoU*) and *IoU*^2^, may be derived by manipulating the parameter in the *α*−*IoU* loss. We can also use multiple *α* values to extend the previous formula to loss functions with multiple IoU components (e.g. RIoU), when *α* = 2.With the above *α*−*IoU* formula, we can now generalize the commonly used IoU-based losses including *L*_*IoU*_, *L*_*GIoU*_, *L*_*DIoU*_, and *L*_*CIoU*_ using the same power parameter *α* for the IoU and penalty terms:

$$L_{\alpha -IoU}=1-IoU^{\alpha_{1}}+P^{\alpha_{2}}(B,B^{gt}),\alpha ↛0$$ (2)

This simple addition provides a simple generalisation of current IoU-based loss functions depending on the value of *α*, which improves the regression accuracy of bbox by adaptively reweighting the loss and gradient of high and low IoU targets, which improves the regression accuracy of bbox. Experiment with a variety of object detection benchmarks to see which ones are more durable in the face of short datasets and noisy boxes. In this research, we employ the Alpha-IoU loss function as the loss function for supervised training on the model. The improvement in detection performance is great, and the network model’s contribution to the final outcome is significant. It was discovered throughout the experiment that Alpha-IoU loss is more friendly to lightweight network performance, and the detection accuracy enhancement effect is more visible.

## Experimental results and discussion

### Implementation details and evaluation metrics

The recommended Alpha-SGANet is built with the Pytorch1.8 library and trained on a single GeForce RTX 3090Ti GPU from start to finish. The models are trained for 300 epochs in total, including a 5-epoch warmup. We employ stochastic gradient descent for training (SGD). We use a learning rate of *lr*×BatchSize/16, with an initial *lr* = 0.01 and the cosine schedule. The SGD momentum is 0.98, while the weight decay is 0.001. We use the Pascal VOC and COCO datasets because they are popular for similar tasks, and we conduct all ablation experiments on Pascal VOC, with both datasets being used for the final comparison with other state-of-the-art lightweight object identification models in the literature. There are 16,551 photos in the Pascal VOC, which covers 20 categories and an average of 2.4 bounding boxes each image. COCO is a larger dataset with 117,264 pictures, including 80 object classes, and 7.4 bounding boxes per image on average. For training, we combine the Pascal VOC 2007 and 2012 training datasets and compare performance on the 2012 test split. We use the COCO 2017 dataset’s train and validation splits for COCO. We utilize the default IoU threshold of 0.5:0.95 for Pascal VOC, and other detailed metrics suggested by the dataset for COCO. The experiment will compare mAP size, network structure characteristics, floating-point operations (GFLOPs), and real-time detection speed FPS (Frames Per Second) using a set of weight files with the lowest loss in each round. It takes about a day to train once, and 2 to 3 days to train on the COCO dataset.

The following is definitive of IoU, We used the standard assessment metrics mAP:

$$IoU=\frac{\mathrm{area}(B_{p}\cap B_{gt})}{\mathrm{area}(B_{p}\cup B_{gt})}$$ (3)

Where *B*_*p*_ denotes the detection network’s prediction bounds and *B*_*gt*_ denotes the Ground-Truth body region. We utilized the average IoU of all the test photos. The following is the AP’s clear statement:

$$AP=\frac{1}{N}\sum_{i=0}^{\mathrm{all}}\frac{TP}{\left(TP+FP\right)}$$ (4)

The true positive is denoted by TP, while the false positive is denoted by FP. And N denotes the detection result’s numbers. The average value of all categories is denoted by AP. This is known as "mean average precision" (mAP), and the mAP utilized in this article is the same as that used in the MS COCO data set, which employs 10 IoU thresholds of 0.50:0.05:0.95.

### Results on PASCAL VOC

#### PASCAL VOC

Natural photos from 20 classifications make up the dataset. Our Alpha-SGANet is trained on the union set of VOC 2007 and VOC 2012 trainval, and we report single-model results on the VOC 2007 test with a 640×640 input size.

The proposed model is compared with the network YOLOv5s, YOLOv5m, YOLOv5l, YOLOv5x of four versions of YOLOv5, as shown in Table 1. And YOLOv5s-Ghost and YOLOv5s-MobileNetv3 were generated using GhostNet and MobileNetv3 as the backbone network of the original YOLOv5s respectively, and they were also included in the comparison. When the input size is the same as 640×640, the comparison of parameter quantity, speed and accuracy is shown in Table 1.

**Table 1:** Performance comparison of each model on VOC data.

	Input size	Parameters	mAP%	FPS
YOLOv5s	640*640	7.27	57.62	128.2
YOLOv5m	640*640	21.13	60.31	93.46
YOLOv5l	640*640	46.73	62.86	67.11
YOLOv5x	640*640	87.37	64.70	54.35
YOLOv5s-Ghost	640*640	5.10	51.30	122.49
YOLOv5s-MobileNetv3	640*640	3.59	43.20	116.42
Alpha-SGANet	640*640	4.93	**65.14**	68.49
Alpha-SGAsNet	640*640	2.84	62.62	105.3

The accuracy of the two models developed by attempting to merge the lightweight network with the backbone network has decreased, as shown in Table 1, while the speed of the two models has improved slightly. In terms of accuracy and speed, the results obtained by combining YOLOv5s-MobileNetv3 decreased by nearly 9.5% mAP, whereas the results obtained by combining MobileNetv3 are not as good as the results obtained by combining GhostNet, demonstrating that GhostNet can effectively generate high-quality feature maps in this case. The technique Alpha-SGANet proposed in this work obtains the highest accuracy of 65.14% mAP with a parameter amount of 4.93 MB, which is 7.52% higher than the 57.62% mAP of the original YOLOv5s and 0.44% higher than YOLOv5x. 68.49 FPS is also the real-time frame rate. The speed is minimal when compared to the original YOLOv5s’ 128.2 FPS, but the accuracy improvement is remarkable. Furthermore, our starting point was to comprehensively weigh the two indicators of speed and accuracy. We did not add the P6 architecture because the speed is only 68.49 FPS; however, its speed has been greatly improved, up to 105.3 FPS, and its accuracy of 62.62% mAP has increased by 5% mAP when compared to the original YOLOv5s; and its model is largely lightweight, with only 2.84 MB model parameters. This result is closer to our original design objective, allowing us to achieve a better balance of speed and accuracy, as well as the ability to run models on embedded platforms and edge devices, which is crucial in engineering practice.

**Fig 8:**
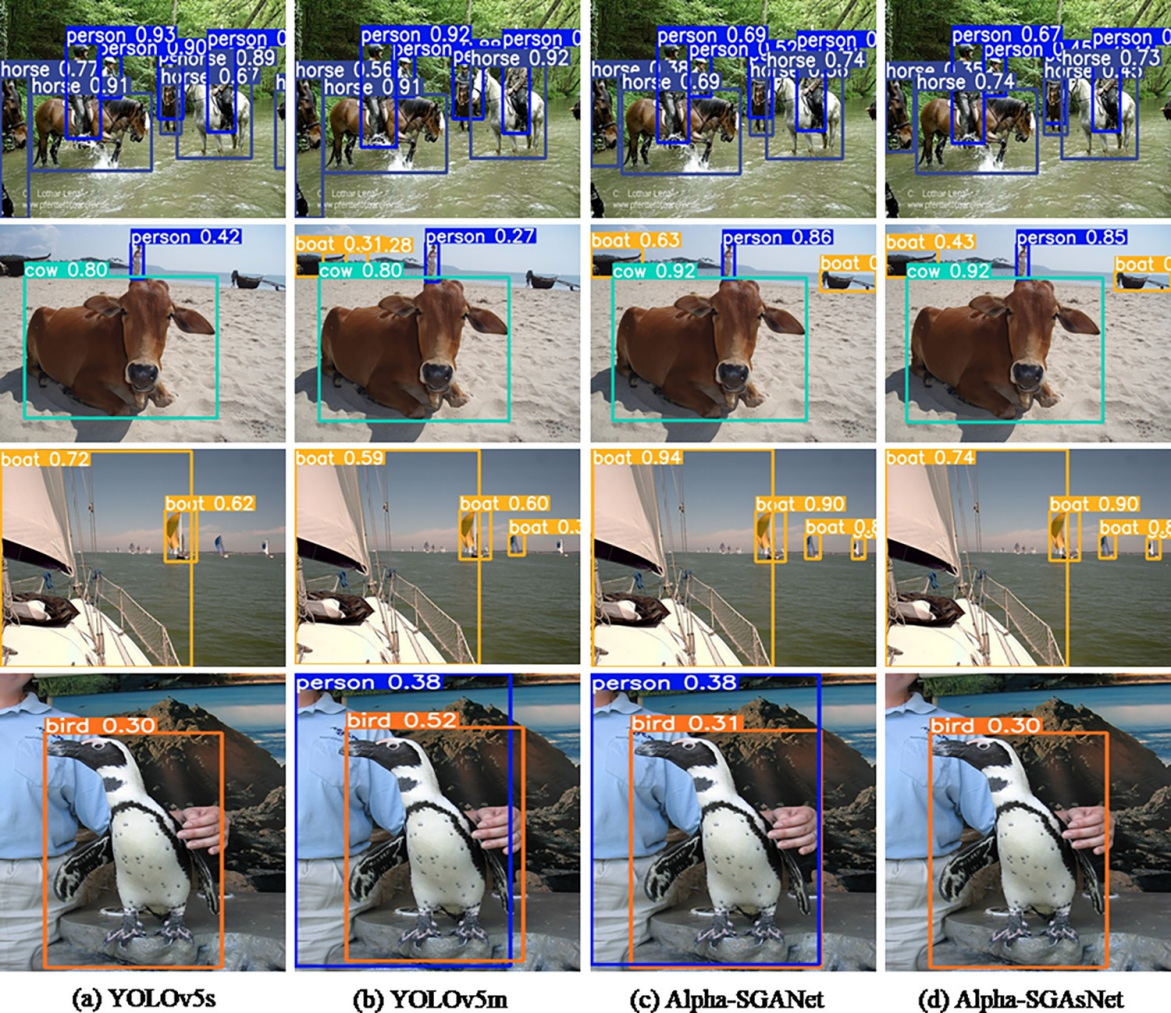
On the Pascal VOC 2007 test dataset, some visual comparisons to different baselines. The equivalent detection outputs from YOLOv5s, YOLOv5m, Alpha-SGANet, and Alpha-SGAsNet are shown from left to right. Various colored enclosing boxes denote various categories. The category and its recognition confidence are referenced in the upper left corner of the bounding box.

Fig 8 shows some visual comparisons of detection outputs from different algorithms using the PASCAL VOC 2007 test dataset. From left to right, the corresponding detection outputs from YOLOv5s, YOLOv5m, Alpha-SGANet, and Alpha-SGAsNet are shown.

Although YOLOv5s and YOLOv5m can efficiently detect horses and persons on horseback in the first row, Alpha-SGANet and Alpha-SGAsNet are still in the bounding box when the object is quite crowded. Boundary regression is more precise and can address edge details better. The image in the second row depicts both large and small targets, such as animals and distant humans and ships. When two targets with considerable size disparities occur at the same time, Alpha-SGANet and Alpha-SGAsNet are able to successfully cope with the issue and detect all of the targets in the image, however, YOLOv5s and YOLOv5m have missed detection and are unable to detect faraway ships. Similarly, Alpha-SGANet and Alpha-SGAsNet demonstrate high robustness and can efficiently adapt to the obstacles posed by size changes in scene detection of the same target in the third row. Alpha-SGANet, on the other hand, uses four feature scale fusions and Alpha-well-designed SGAsNet’s outcomes, whilst YOLOv5s and YOLOv5m have missed detections, although YOLOv5m performs better. Both a penguin and a person standing behind them are depicted in the final row of images. While YOLOv5s can detect penguins precisely, it ignores the people in their wake, leading to missed detection, whereas YOLOv5m can detect both penguins and people simultaneously. Despite their being just two objects, there are some evident instances where the bounding box is incorrect, such as the human hand and the penguin’s jaw tip, which are not very well framed on the boundary. The regression on the edge features of the bounding box is more precise when using Alpha-SGANet, which can simultaneously recognize a penguin and a person standing behind it as targets. Alpha-SGAsNet has a more precise bounding box even though it only detects one target of penguin. All of the data suggest that the proposed method is robust in crowded scenes, scenes with substantial target size variances, scenes with the same target size moving, and large-scale target identification scenarios, and that it can be applied to a variety of detection demands in varied situations.

### Results on MS COCO

#### MS COCO

Natural pictures from 80 different object categories make up the dataset. We use trainval35k for training, minival for validation, and test-dev for single-model findings, as is standard practice.

As shown in Table 2, our proposed model is compared with other state-of-the-art object detectors. First compared with YOLOv5s and YOLOv5m, when the same input image size is 640×640, Alpha-SGANet achieves 39.73% mAP value with 5.17 MB of parameters, which is 4.13% higher than YOLOv5s accuracy. It can be reduced, and the speed can also be achieved in real-time. Alpha-SGAsNet achieved an accuracy of 37.84% mAP with a parameter size of 3.01 MB, and a real-time speed of 104.2 FPS, which was only slightly reduced compared to the 114.9 FPS of YOLOv5s. Since we chose YOLOv5s as the benchmark model, it is slightly less than the 43.1% mAP of YOLOv5m, but the parameter size of YOLOv5m is as high as 21.37 MB, which is almost 4 times that of Alpha-SGANet and 7 times that of Alpha-SGAsNet. Compared with YOLOX-s, the accuracy is more comparable in the case of fewer parameters. As for YOLOX-Nano and YOLOX-Tiny, compared with our proposed model, it is even less advantageous. Compared with the newly proposed lightweight network PP-PicoDet, we propose that Alpha-SGAsNet has better performance than PP-PicoDet-S and PP-PicoDet-M, but slightly less than PP-PicoDet-L. In addition, we also compared some existing classic lightweight network models, such as PeleeNet [47], NanoDet [48], EfficientDet [49], YOLOv4, and PP-YOLO-Tiny, etc. In terms of parameters and parameters, we suggest Alpha-SGANet and Alpha-SGAsNet. The accuracy and even the speed are comparable and can achieve good results to varying degrees, demonstrating the efficacy of our suggested strategy, which can successfully minimize the number of model parameters while maintaining speed and accuracy. It adds significant value to engineering practice when deployed on platforms with low computer capabilities, such as embedded.

**Table 2:** Comparison of the state-of-the-art model on MS COCO data.

Model	Size	Params	GFLOPs	mAP%	FPS
PeleeNet	304*304	6.0	1.29	22.4	-
CSL-YOLO	512*512	3.2	2.22	26.3	-
MobileNet-SSDLite	320*320	4.3	0.8	22.1	-
NanoDet-M	320*320	0.95	0.72	20.6	-
NanoDet-M	416*416	0.95	1.2	23.5	-
NanoDet-M-1.5x	416*416	2.08	2.42	26.8	-
EfficientDet-D0	512*512	3.9	2.5	33.8	98.0
EfficientDet-D1	640*640	6.6	6.1	39.6	74.1
RetinaNet-R50	640*640	34	97	39.2	-
RetinaNet-R101	640*640	53	127	39.9	-
PP-YOLO-Tiny	320*320	1.08	0.58	20.6	-
PP-YOLO-Tiny	416*416	1.08	1.02	22.7	-
YOLOX-Nano	416*416	0.91	1.08	25.3	-
YOLOX-Tiny	416*416	5.06	6.45	32.8	-
YOLOX-s	640*640	9.0	26.8	39.6	102.0
PP-PicoDet-S	320*320	0.99	0.73	27.1	-
PP-PicoDet-S	416*416	0.99	1.24	30.6	-
PP-PicoDet-M	416*416	2.15	2.50	34.3	-
PP-PicoDet-L	640*640	3.30	8.91	40.9	-
YOLOv3	608*608	61.94	156.3	33.0	52.0
YOLOv3-SPP	606*608	62.99	157.1	36.2	73.1
YOLOv3-Tiny	416*416	8.85	13.2	17.3	357.1
YOLOv3+ASFF	320*320	-	-	38.1	60.0
YOLOv3+ASFF	416*416	-	-	40.6	54.0
YOLOv4	416*416	64.36	35.5	41.2	96.1
YOLOv4-Tiny	416*416	6.1	6.9	21.7	371.2
YOLOv5s	640*640	7.2	17.1	35.6	114.9
YOLOv5m	640*640	21.37	51.4	43.1	95.23
Alpha-SGANet	640*640	5.17	7.0	39.73	74.6
Alpha-SGAsNet	640*640	3.01	7.3	37.84	104.2

**Fig 9:**
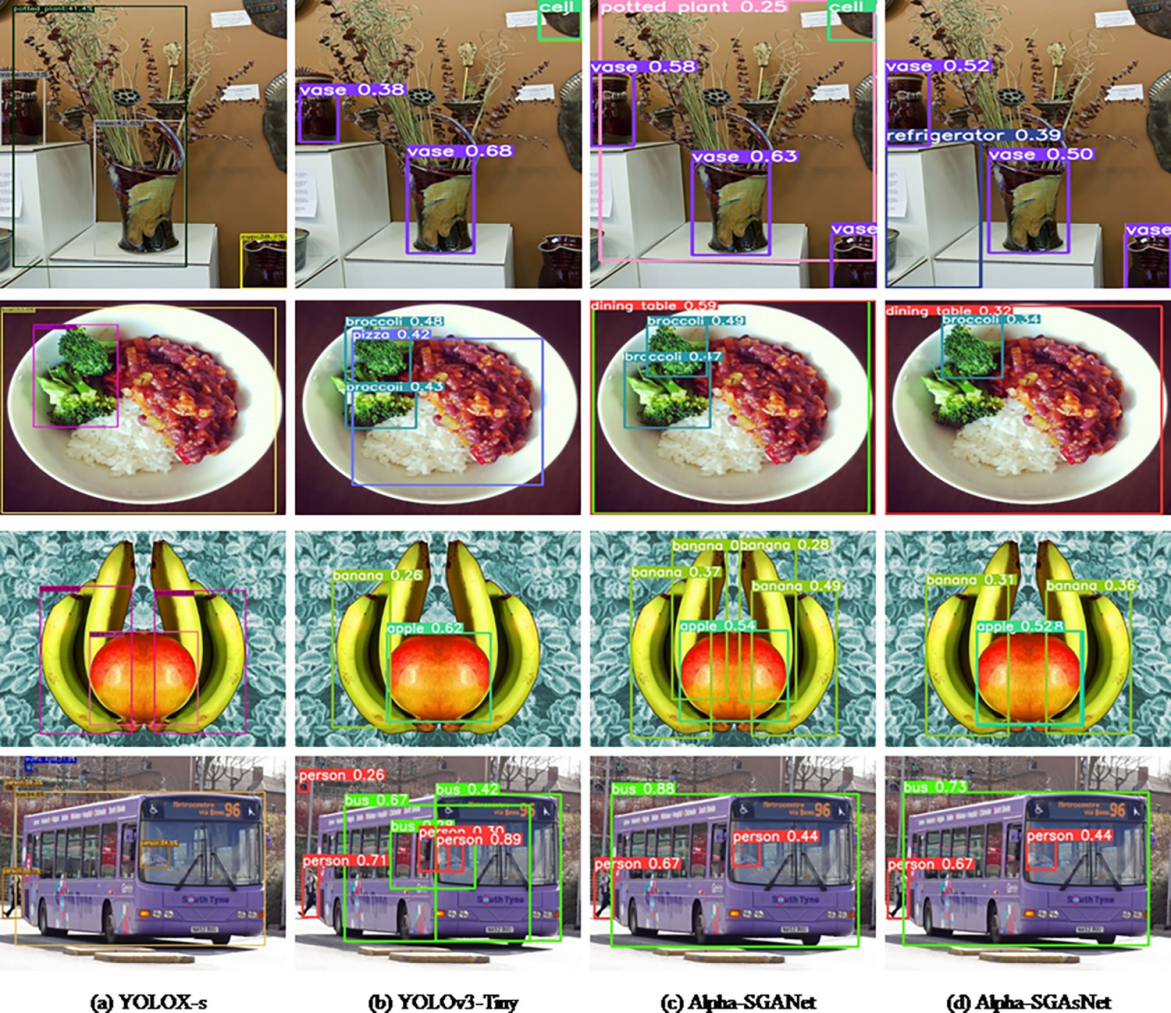
On the MS COCO test dataset, some visual comparisons to various baselines. The equivalent detection outputs from YOLOX-s, YOLOv3-Tiny, Alpha-SGANet, and Alpha-SGAsNet are shown from left to right. Various colored enclosing boxes denote various categories. The bounding box’s upper left corner corresponds to the category and its recognition confidence.

In Fig 9, we also provide some visual examples of detection outputs from additional algorithms on the MS COCO test dataset. The detection results from YOLOX-s, YOLOv3-Tiny, Alpha-SGANet, and Alpha-SGAsNet are displayed in the first to fourth columns, respectively. In the detection comparison of the first line of pictures, the results detected by YOLOX-s are not particularly precise on the edge of the bounding box and do not entirely contain all the plants and the center vase, despite the fact that it can simultaneously detect all vases and potted plants. The selection of the frames is not particularly precise. The detect result of the middle vase by YOLOv3-tiny is more correct, but it skips over the vase and potted plants in the lower right corner of the image, skipping inspections. With respect to the first two algorithms, Alpha-SGANet performs well. The edge processing of the target frame is more accurate while simultaneously detecting all vases and potted plants. Another Regression on the target item performs better even though Alpha-SGAsNet failed to recognize potted plants. While the other two comparison algorithms have imprecise positioning in the second row of comparison images, Alpha-SGANet and Alpha-SGAsNet are able to more precisely detect the border of the vegetables in the bowl for selection. Bananas and pomegranates can both be found in the third row of the images. All four algorithms have produced false detections due to how similar pomegranates and apples look. The border pays more attention to details, while Alpha-SGANet is more effective in accurately detecting all bananas in terms of banana detection performance. Despite the fact that all three algorithms include Alpha-SGAsNet, YOLOX-s, and YOLOv3-Tiny have missed detections, Alpha-SGAsNet is more precise and performs better in the regression of bounding boxes. In comparison to YOLOX-s and YOLOv3-Tiny, there is a false detection and the frame selection region is incomplete when detecting the bus. Alpha-SGANet and Alpha-SGAsNet are better at adapting to changes in target scale, framing the bus in the image, and framing the image of a person. Such a minor goal, but one that is the outcome of our network’s careful design. According to the results, our method can capture context information more precisely, and this dataset requires more rigorous edge processing of the bounding box.

### Ablation study

The findings of ablation research are reported in Table 3 since the model in this work integrates numerous modules to prove the necessity of these modules for the final model. Because training takes time, this research opts to conduct ablation experiments on the PASCAL VOC data set using some of the newly introduced modules or approaches. During the experiment, the input images are 640*640 pixels, and the reference is YOLOv5s. The results are presented in Table 3.

**Table 3:** Ablation study on PASCAL VOC set.

	P6	Ghost	CBAM	SFNet	L_α−IoU_	Paras	GFLOPs	mAP%	FPS
YOLOv5s						7.27	17.20	57.62	128.2
OurNet	**√**					12.43	16.90	59.83	91.74
OurNet	**√**	**√**				6.77	8.70	53.91	97.60
OurNet	**√**	**√**	**√**			6.89	8.50	57.60	60.81
OurNet	**√**	**√**	**√**	**√**		5.04	6.60	56.60	68.96
OurNet	**√**	**√**	**√**	**√**	**√**	4.93	6.50	65.14	68.49
OurNet		**√**	**√**	**√**	**√**	2.84	6.80	62.62	105.3

As shown in Table 3, we use YOLOv5s as the reference benchmark model and simply extend it to four feature scales for detection, that is, when adding one more layer of downsampling P6, the model accuracy is improved from 57.62% mAP to 59.83% mAP, the mAP value was increased by 2.21%, and the number of parameters increased a little as expected, from 7.27 MB to 12.43 MB, and the model inference speed also slowed down as we expected, from 128.2 FPS to 91.74 FPS. The addition of the P6 downsampling layer adds a layer of calculation and complexity, slowing model inference speed, and the increase in the number of parameters is not conducive to edge device deployment but may perform well on the PC side. Resources may impose a limit on the speed. We initially assumed it would result in a large speed boost, but the real speed boost isn’t noticeable when compared to the original P6-based network. 91.74 FPS, an increase of 5.86 FPS, and a 5.92% mAP drop in accuracy, which is reasonable. The number of parameters and floating-point operations has been significantly reduced, which is reduced by half compared to P6. It can be seen that the addition of the lightweight network GhostNet reduces the number of parameters and increases the inference speed, but the accuracy is reduced. Based on the two, continue to add the CBAM attention mechanism module to the neck part, the parameters are slightly increased, but the accuracy is increased from 53.90% mAP to 56.70% mAP. The experiment proves that the addition of the CBAM module can hardly increase the parameters. The accuracy of the model can be effectively improved in the case of large quantities, but it may be related to the structure of the module itself or the location of the module added, resulting in a slower model inference speed, only 60.81 FPS, which is contrary to the original intention of our design. Faced with such a situation, we tried to combine a lighter ShuffleNetV2 module in the backbone part. The addition of the ShuffleNetV2 module can increase the speed from 60.81 FPS to 68.96 FPS while reducing the accuracy by only 1% mAP while reducing the number of parameters by 1.85 MB. Finally, we tried to use Alpha-IoU Loss to generate our final model Alpha-SGANet, and obtained the highest accuracy of 65.14% mAP. Its model parameters are only 4.93 MB, and the real-time speed is 68.49 FPS. It can be seen that Alpha-IoU loss can be not in speed. In the case of influence, it will bring a huge improvement to the model accuracy, and the improvement effect is better in the case of a lightweight network model, which almost brings an improvement of 8.54% mAP value, which is higher than the original YOLOv5s 57.62% mAP 7.52% mAP. However, the initial starting point is to improve the accuracy and the speed of the model and at the same time reduce the number of model parameters, so we remove the P6 layer here and get a lighter weight that can take into account speed and accuracy at the same time. A small model version of the network SGAsNet. The number of model parameters is only 2.84 MB, and the model accuracy has a value of 62.62% mAP, which is 5% mAP higher than the original YOLOv5s, and the real-time inference speed of the model is 105.3 FPS. Compared with the original YOLOv5s, it is only slightly slower. It can get the best trade-off in terms of speed and accuracy, and the parameter amount of the model is only 2.84 MB. Such a network model can be easily deployed to resources on restricted embedded platforms or edge mobile devices.

During the experiment, we attempted combining SE attention in the neck region of the model, CA attention module, and CBAM attention module. The Table 4 displays the results for accuracy comparison and improvement in detail. Additionally, in several well-known references, the CBAM attention module with the YOLOv5 network structure can produce the best accuracy outcomes. As shown in Table 4, the table illustrates how applying the new model we suggested to try to merge the attention module can significantly increase the model’s detection accuracy. The SE module adds a modest number of additional parameters, which slows down the process of increasing the model’s performance. The CBAM module can help maximize the performance of the model and the speed is essentially equivalent to the speed attained by adding the CA attention module. Both modules are small and bring similar amounts of parameters.

**Table 4:** Performance comparison of adding different attention mechanism modules.

Attention module	Input size	Parameters	mAP%	FPS
YOLOv5s	640*640	7.27	57.62	128.2
Ours+SE	640*640	4.31	63.51	94.6
Ours+CA	640*640	4.86	64.79	71.4
Ours+CBAM	640*640	4.93	65.14	68.5

### Speed test result on Jetson AGX

The number of multiply-accumulates (FLOPs) is a common approach to estimating processing costs. It cannot, however, replace a speed test on real devices because many other aspects, such as cache, I/O, hardware optimization, and so on, might affect the actual time cost. This section evaluates the performance of efficient models to demonstrate the efficacy of the method proposed in this research and to further test the proposed algorithm’s actual performance in real-world circumstances on the NVIDIA Jetson AGX Xavier embedded platform. The speed is determined by the average processing time of 400 images with a batch size of one. We put 400 images through the processing pipeline five times and average the results.

For the four versions of YOLOv5, YOLOv5s, YOLOv5m, YOLOv5l, YOLOv5x, and our proposed Alpha-SGANet and Alpha-SGAsNet, the model weights trained on RTX 3090 were transplanted to the embedded platform Jetson AGX for speed testing, During the test, the image size is uniformly set to 640×640, as shown in Table 5. First of all, for the test results of the VOC dataset, it can be seen that YOLOv5s achieved a real-time speed of 19.31 FPS with an accuracy of 57.62% mAP and a delay of 51.8 ms. This performance shows the ingenuity and powerful performance of the YOLOv5 network design, while YOLOv5 and the other three models slowed down almost exponentially with the gradual increase in the accuracy and the number of parameters. In contrast, the Alpha-SGANet proposed in this paper has greatly improved the accuracy with a slight increase in speed, achieving a real-time speed of 19.46 FPS with 65.14% mAP and a delay of 51.4 ms. This also shows that the convolution method of the lightweight network has better performance on the embedded platform than the traditional standard convolution method, while the small model version Alpha-SGAsNet is close to the accuracy of YOLOv5l, and the speed is greatly improved, 62.62% mAP was obtained with 2.84 MB parameters on Jetson AGX, and the real-time speed was 24.23 FPS, which was 4.92 FPS higher than that of YOLOv5s. This result is more in line with our initial design concept. Compared with most existing lightweight networks, it has certain comparability and can meet the real-time detection requirements of many scenarios. In addition, to compare the test results on the VOC and COCO datasets, we also tested on the MS COCO test set. With a slight increase in the number of parameters, the speed of the method proposed in this paper is reduced by about 1 FPS.

**Table 5:** Speed on NVIDIA Jetson AGX Xavier embedded platform.

VOC Data					COCO Data			
	Params	mAP	Latency	FPS	Params	mAP	Latency	FPS
	(M)	(%)	(ms)		(M)	(%)	(ms)	
YOLOv5s	7.27	57.62	51.8	19.31	-	-	-	-
YOLOv5m	21.13	60.31	117.6	8.50	-	-	-	-
YOLOv5l	46.73	62.86	205.9	4.85	-	-	-	-
YOLOv5x	87.37	64.70	394.8	2.53	-	-	-	-
Alpha-SGANet	4.93	65.14	51.4	19.46	5.17	39.73	53.1	18.83
Alpha-SGAsNet	2.84	62.62	41.1	24.23	3.01	37.84	43.7	22.88

## Conclusion

In this research, we propose the Alpha-SGANet lightweight network, which has greatly enhanced accuracy and can match real-time requirements when compared to YOLOv5s. On the PASCAL VOC dataset, the accuracy is as high as 65.14% mAP, with a real-time speed of 68.49 FPS, and on the MS COCO dataset, the accuracy is as high as 39.73% mAP, with a real-time speed of 74.6 FPS, using the NVIDIA GeForce RTX3090 GPU platform. The suggested tiny model version Alpha-SGAsNet achieves a comparable available trade-off in terms of detection accuracy and implementing efficiency on PASCAL VOC and MS COCO datasets for a better trade-off between speed and accuracy. Get 62.62% mAP with 2.84 MB of parameters and 105.3 FPS in real-time on the PASCAL VOC dataset, and 37.84% mAP with 3.01 MB of parameters and 104.2 FPS in real-time on the MS COCO dataset. In contrast to top accurate networks with complicated and deep architectures that are computationally expensive, our Alpha-SGAsNet focuses on constructing a lightweight network backbone and strong bounding box regression accuracy, achieving a single trade-off between accuracy and efficiency. Alpha-SGANet has a real-time speed of 19.46 FPS, and Alpha-SGAsNet has a real-time speed of 24.23 FPS, which is 4.92 FPS greater than the 19.31 FPS of YOLOv5s, as confirmed by merging the VOC dataset on the Nvidia Jetson AGX platform. On embedded platforms, this outcome can meet the real-time needs of most scenarios. There are still significant issues with the method suggested in this research, despite some of its advantages. Firstly, the approach used in this paper is a novel technique to combine various approaches or modules. Due to the clever design of this paper, it is possible to achieve a certain performance improvement when the number of model parameters is significantly reduced for natural scene detection, but the method this paper suggests is not the best one. It takes scientific design approaches and several tries to figure out how to construct and optimize the network more thoroughly from the input, backbone, neck, detection head, and output of the network structure according to the requirements of the scene. Additionally, the combination of Alpha-IoU loss used in this paper can assist in returning a more accurate bounding box, which can effectively improve the detection accuracy when the IoU threshold is set larger. As a result, the comprehensive mAP (0.5:0.95) will be significantly improved, but it was discovered during the experiment that the single mAP (0.5) accuracy index’s improvement effect is not immediately apparent. How to make targeted improvements to the Alpha-IoU loss that will allow it to be perfectly applied to any IoU threshold and lead to a qualitative improvement in model detection performance. Finally, although the technology described in this study can execute at a certain speed on an embedded platform, the improvement effect is not substantial. In the future, we can try to develop a network topology more suited for embedded devices and try to merge techniques like TensorRT to help further enhance the speed.

## Supporting information

S1 Dataset The authors declare that provided the minimal dataset of output detection for all algorithms taking part in the comparison in the visualization results presenting section of this publication in order to illustrate the genuine validity of this paper. (ZIP)
